# Exploring Age, Gender and Pandemic Dynamics: A Retrospective Analysis of the Impact of SARS-CoV-2 on Human Health

**DOI:** 10.3390/life15030355

**Published:** 2025-02-24

**Authors:** Diana Vrabie, Beatrice-Aurelia Abalașei, Marius Neculăeș

**Affiliations:** Faculty of Physical Education and Sport, University Alexandru Ioan Cuza of Iași, 700506 Iași, Romania; abalasei.beatrice@uaic.ro (B.-A.A.); marius.neculaes@uaic.ro (M.N.)

**Keywords:** COVID-19 incidence, Romania, Europe, life expectancy

## Abstract

Background: The deaths due to the SARS-CoV-2 virus increased rapidly over a short period of time. From the beginning of the pandemic until September 2024, the number of COVID-19 cases reached 776,205,140 cases, with 7,064,380 deaths worldwide. The total number of deaths recorded from the beginning of the pandemic until September 2024 represents a share of 0.09% of the total world population and 0.91% of the total infected population. Methods: The data in this study were collected from the Ministry of Internal Affairs of Romania, Eurostat, NIS and WHO sites, and analyzed using Microsoft Excel and SPSS 22 in order to detect the evolution trends during the state of emergency, the share of infection and deaths among the European countries and worldwide, differences between gender categories or age groups and to identify factors that can be related to the incidence of infection and mortality due to COVID-19. Results: Males registered a significant higher number of deaths compared to women in Romania, a trend that aligns with global tendencies (*p* < 0.05). Also, the 70–79 age group had the highest mortality rate, followed by the over 80 age group and the 60–69 age group. Therefore, the risk of death from COVID-19 increases significantly with age. A good health education may be essential in order to promote a high life expectancy. A higher life expectancy correlated with a lower rate of mortality. BMI can be a factor contributing to the increased comorbidities, and may influence the fatality levels of COVID-19.

## 1. Introduction

The World Health Organization declared a global pandemic situation on 11 March 2020. After analyzing the data reported by the WHO, we concluded that the percentage of infected people worldwide increased from 2% in April 2021 to 10.44% in September 2024. The increasing share may reflect not only an increase in the number of infections, but also factors such as an increased testing frequency and the possibility of multiple testing of the same person. Therefore, changes in testing strategies and the wide access to tests have contributed to a more accurate SARS-CoV-2 identification, but also made the share of infections not directly correlated with an overall infection rate.

On Monday 16 March 2020, Romania also declared a state of emergency. On 10 June 2020, the total number of infections was 20,945, with 1360 deaths nationwide. From the beginning of the pandemic until September 2024, in Romania, 3,563,055 cases of infection with SARS-CoV-2 were registered, alongside 68,893 deaths caused by COVID-19. The share of people infected with SARS-CoV-2 among the Romanian population increased from 5.37% in April 2021 to 17.16% in March 2023 and 18.24% in September 2024. The share is calculated from the total population, checking the periodic increase in the numbers of infections reported by WHO. The percentage of deaths from total population increased from 0.14% (April 2021) to 0.34% (March 2023) and 0.35% (September 2024).

Of the total number of infected people, 1.93% died due to the COVID-19 disease in Romania from the start of pandemic until 26 September 2024.

**Figure 1:**
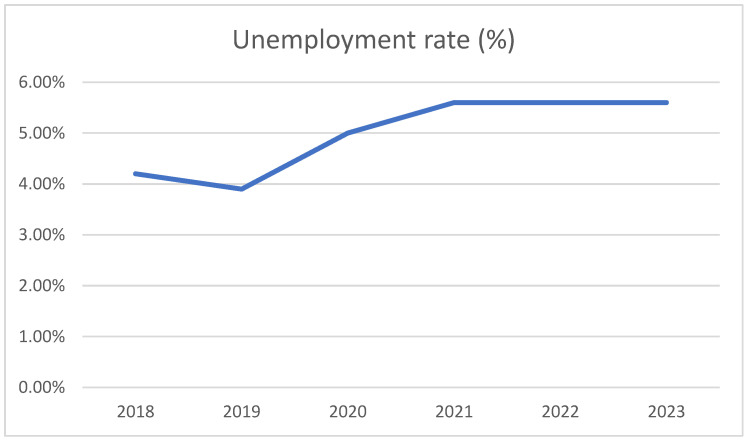
Unemployment rate in Romania, 2018–2023.

The COVID-19 pandemic has had a major impact on public health worldwide, affecting not only health systems but also economies, education and social life. Figure 1 shows the evolution of unemployment rates in Romania during the 2018–2023 period. As can be seen, 2021 marked an increase in the unemployment rate, which remained constant in the following 3 years.

**Figure 2:**
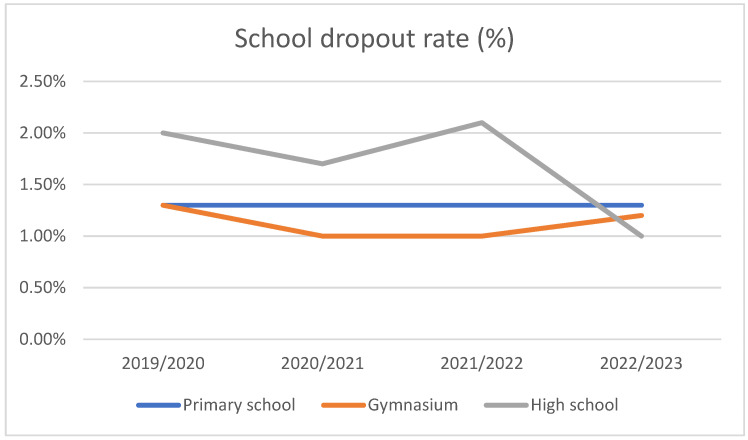
School dropout rate, 2019–2023.

Regarding education, Figure 2 shows the school dropout rate during the 2019–2023 period. Particularly the high-school students registered a peak of school dropout in 2021, with 2.3% of high school students withdrawing.

In Romania, as in many other countries, the virus caused a health crisis, prompting the authorities and public health institutions to adopt measures to prevent and combat the virus spread.

The purpose of this study is to analyze the health of the Romanian population before, during and after the COVID-19 pandemic, exploring the trends and characteristics of deaths in order to identify vulnerable groups and highlight the differences between the impact of COVID-19 on men and women, as well as between different age groups.

This report aims to analyze the impact of the COVID-19 pandemic on different demographic groups in Romania and Europe, with a focus on the distribution of deaths by age and gender. Despite the research conducted to date, there are still many unexplored aspects. Therefore, this report aims to outline some potential strategies to optimize the national response to potential future health crises, reducing exposure and mortality among vulnerable categories. The analysis is trying to identify the impact of SARS-CoV-2 infection on mortality rate. In order to contextualize the situation from Romania in a wider framework, this study also includes a comparison of the situation in Romania and other European countries.

## 2. Materials and Methods

This report contains data from before, during and after the COVID-19 pandemic. Data related to the annual number of deaths in Romania, the seasonality and causality of deaths and differences between genders were collected from the Romania NIS site. The data including the total number of SARS-CoV-2 infections in Romania, the total number of deaths (cumulative), the 24 h reported deaths number, the number of deceased females/males, the number of deaths by age groups (under 30; 30–39; 40–49; 50–59; 60–69; 70–79; over 80) and the presence/absence of comorbidities were collected from June 2020 to March 2022 (636 days) based on daily reports from the Romanian Ministry of Internal Affairs site. We also collected data from Eurostat and WHO sites, and used Microsoft Excel and SPSS 22 to analyze the trends in human health status during a wider period of time, before, during and after the COVID-19 pandemic. The Shapiro–Wilk test was used to test the normal distribution of the data. The Mann–Whitney U test was used to assess the differences in mortality by gender category, due to the non-uniform distribution of the data. ANOVA Welch analysis was used to detect potential differences between the number of deaths by age category. To highlight the differences between age categories, we used the Games–Howell post hoc test. The study uses the Pearson correlation to analyze the relationship between the share of infections and share of deaths due to COVID-19, and also between life expectancy in European countries and mortality due to COVID-19.

## 3. Results

### 3.1. Mortality Rates in Romania in 2017–2023 Period

**Figure 3:**
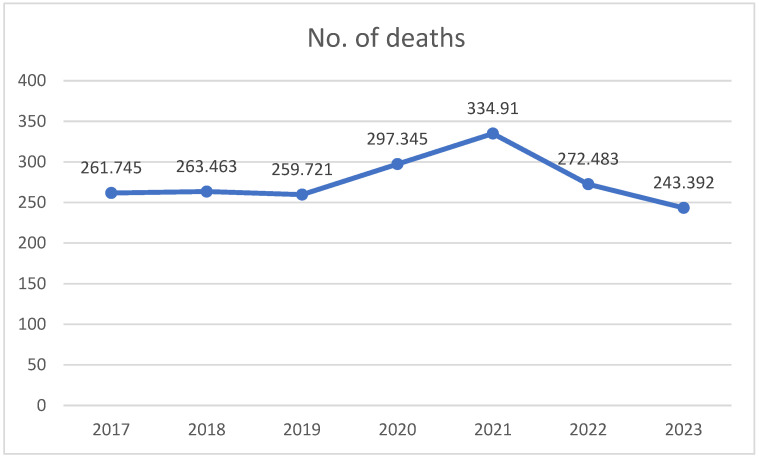
Graphical representation of the number of deaths registered in Romania by year (2017–2023).

Analyzing the evolution of mortality in Romania during 2017–2023, we observed several significant trends in the demographic and causal structure of deaths, directly influenced by the age of the population, but also influenced by the COVID-19 pandemic. According to the NIS of Romania, 2021 was the year with the highest number of deaths in Romania since 1938. This means that, within the borders of Romania, not even in World War II did as many people die as in the second year of the coronavirus pandemic. The highest mortality rate was recorded in 2021, the year that was considered the peak of the COVID-19 pandemic (Table 1 and Figure 3).

**Table 1:** Mortality rate in Romania, 2017–2023.

Year	Deaths Number	Mortality Rate (‰)
2017	261,745	11.8‰
2018	263,463	11.8‰
2019	259,721	11.7‰
2020	297,345	13.4‰
2021	334,910	15.2‰
2022	272,483	12.4‰
2023	243,392	11.1‰

### 3.2. Differences in Mortality Rates Between Genders

**Figure 4:**
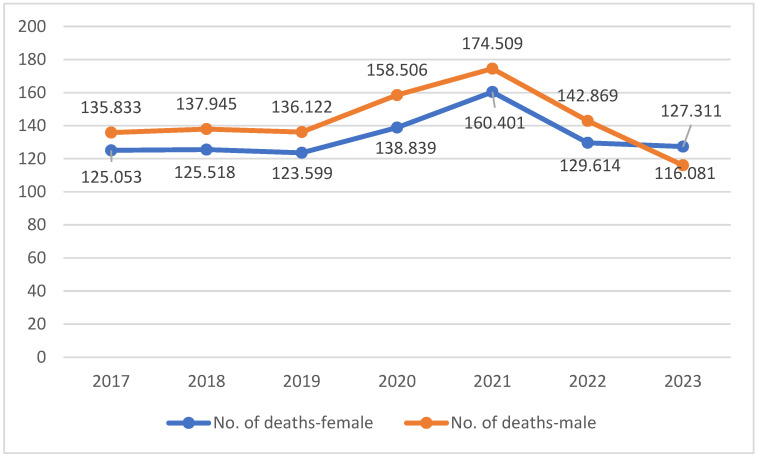
Graphical representation of the deaths by gender (2017–2019).

Between 2017 and 2019, the National Institute of Statistics data show a higher number of deaths among men compared to women (Table 2), except for the 75-and-over age group, where mortality was higher among women. This trend was maintained during the COVID-19 pandemic (Figure 4). A study by Sharma et al. concluded that, worldwide, the risk of death from COVID-19 is 20% higher among men. In addition, male gender has been found to be frequently associated with serious complications requiring intensive care unit admission and mechanical ventilation [1].

**Table 2:** Number of deaths by gender, in Romania, 2017–2023.

Year	Male	Female
2017	135,833	125,053
2018	137,945	125,518
2019	136,122	123,599
2020	158,506	138,839
2021	174,509	160,401
2022	142,869	129,614
2023	116,081	127,311

Given that the risk of death from COVID-19 is strongly related to age and other risk factors for all-cause mortality and also expected remaining life expectancy [2], it is unclear whether the observed sex differences in the COVID-19 fatality rate are simply a reflection of men’s shorter life expectancy [3], which is, at least in part, due to their poorer health status at any given age. The higher fatality rate for men may derive from gender-based immunological differences [4] or can be associated with comorbidities, including hypertension, cardiovascular diseases and drinking alcohol, which are more commonly observed among men [5].

During the state of emergency, between 10 June 2020 and 8 March 2022 in Romania, a total of 64,094 deaths due to COVID-19 were reported. We collected the data from the Internal Ministry of Affairs for 636 days, and we categorized the number of deaths into seven age groups: under 30, 30–39, 40–49, 50–59, 60–69, 70–79 and over 80 years. The deaths were also categorized into two gender groups (male and female).

In order to analyze the distribution of the data, we used the Shapiro–Wilk test on the database with the daily number of deaths by gender. The significance threshold value (*p* < 0.05) indicates that the data do not follow a normal distribution. Thus, we used the Mann–Whitney U test to determine if there are any differences between the two gender categories regarding the number of deaths caused by COVID-19. The result was that males registered a significantly higher number of deaths compared to women (Z = −4.250, *p* < 0.05).

### 3.3. Differences in Mortality Rates Between Age Groups

Most deaths occurred among the elderly, particularly the 75-and-over age group, the group which was consistently responsible for more than half of annual deaths. Since 2020, the pandemic years have marked a significant increase in the number of deaths in this age segment, reflecting the increased vulnerability of the elderly to infections and complications of COVID-19.

During 2020–2021, there were significant increases in deaths in the middle age groups (70–74 years, 65–69 years, 50–54 years), followed by a slight reduction in 2022 and 2023. These fluctuations can correlate with the impact of different waves of the pandemic and with the side effects of sanitary control measures (such as isolation and social restrictions).

**Figure 5:**
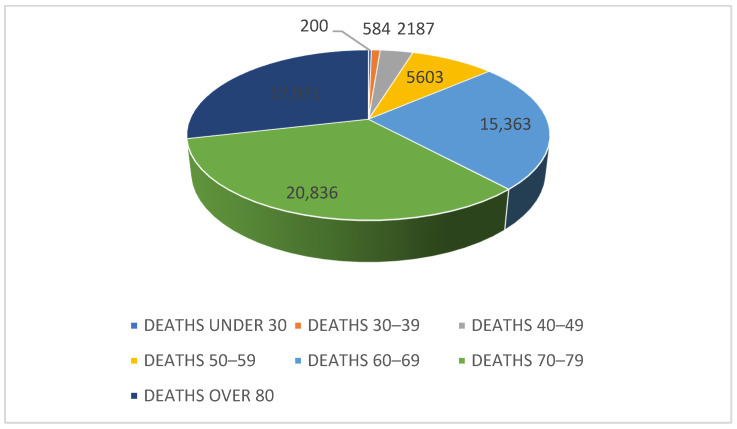
Graphical representation of deaths by age group.

We categorized the number of deaths into seven age groups: under 30, 30–39, 40–49, 50–59, 60–69, 70–79 and over 80 (Table 3). Among these, the 70–79 age group had the highest mortality rate, followed by the over 80 age group and the 60–69 age group (Figure 5).

**Table 3:** Number of deaths by age group in Romania (10 June 2020–8 March 2023).

Age Group	Number of Deaths	Period
Under 30	200	10 June 2020–8 March 2022
30–39	584	
40–49	2187	
50–59	5603	
60–69	15,363	
70–79	20,836	
Over 80	17,971	

In order to analyze the differences between groups, first of all we tested the homogeneity of the variance and normal distribution. The results of the Levene test (*p* < 0.05) suggest that there is no homogeneity of variance between the age groups. The results of the Shapiro–Wilk test suggests that the data do not follow a normal distribution. Because the data do not follow a normal distribution and the homogeneity of the variance is not equal, we used the ANOVA Welch test. The results of the ANOVA Welch test showed that there are significant differences between the age categories in terms of the number of deaths caused by COVID-19 (*p* < 0.001). Thus, we can conclude that age can influence the number of deaths, and the observed differences between age categories are much greater than the internal variability within each category.

The Games–Howell post hoc test confirmed the differences between age groups and identified the following aspects: Significant differences were identified between most age groups, excepting a few specific comparisons, and no significant differences were observed between the 60–69 group, the 70–79 group and the over 80 age group (*p* > 0.05).

We can conclude that older age groups (60–69 years, 70–79 years, over 80 years) did not register significant differences between them, but had a significantly higher number of deaths compared to younger age groups (under 30 years, 30–39 years, 40–49 years), confirming that older age can be an important risk factor of death due to COVID-19.

In conclusion, the ANOVA Welch and Games–Howell post hoc analyses suggests that the risk of death from COVID-19 increases with age, with no differences between higher age groups, but notable differences between younger and older age groups. These differences emphasize the importance of age as a determining factor in the severity of COVID-19. These findings also imply that public health and prevention efforts should consider age when developing protection and intervention strategies, as the impact of COVID-19 differs across age groups.

### 3.4. Causality and Seasonality of Deaths in Romania, 2017–2023

**Figure 6:**
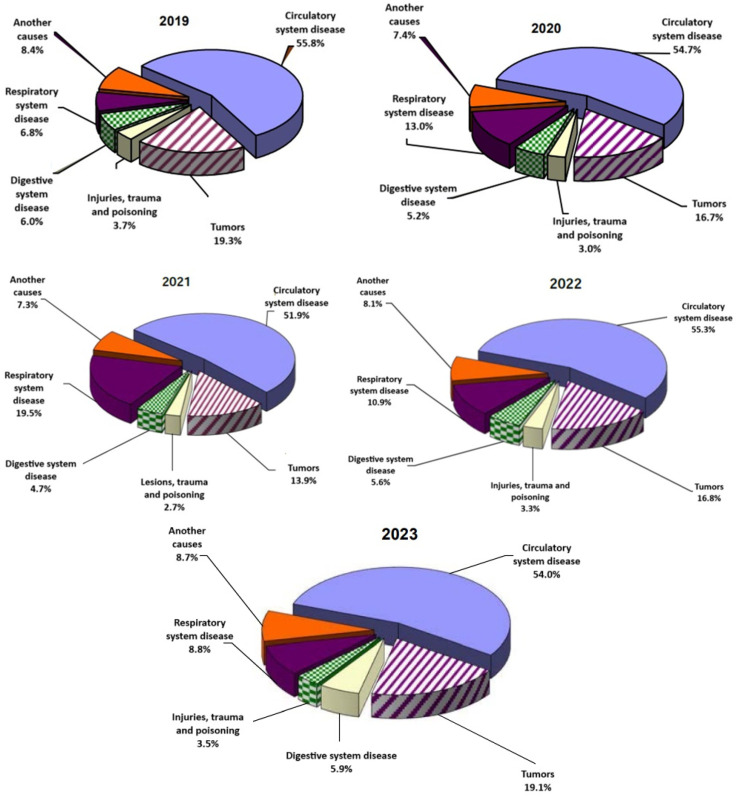
The causes of deaths in Romania, 2019–2023. image from the NIS site, translated (accessed on 22 January 2025).

Regarding the cause of death, the pandemic also had a significant impact on this trend. In 2020, deaths from respiratory diseases increased considerably, from 6.8% in 2019 to 13.0%, which was directly related to the increased incidence of COVID-19. After a peak in 2021 (19.5% from total population), the number of deaths due to respiratory diseases declined in 2022 (10.9%), a fact that can be attributed to a decrease in COVID-19 infections (Figure 6). In the pre-pandemic period, diseases of the circulatory system, including ischemic heart disease and cerebrovascular disease, were the most common causes of death, followed by tumors and respiratory diseases. During the pandemic, the share of deaths caused by respiratory diseases increased significantly, highlighting the huge impact of COVID-19 on the respiratory system.

**Figure 7:**
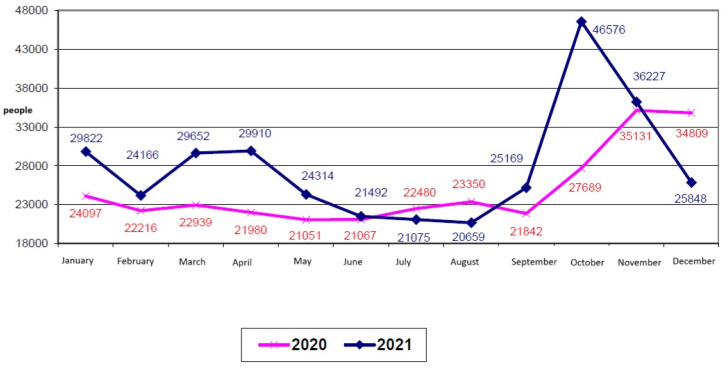
Deaths by month, 2021 compared to 2020, image from the NIS site, translated (accessed on 22 January 2025).

Regarding the seasonality of deaths in Romania, according to the NIS, the number of deaths has varied significantly over the years, with a maximum number of deaths in the winter months, especially in January and November, and a minimum number in the summer months. This is a general trend, but it was accentuated in 2021, when the autumn months, especially October, saw significant increases compared to the previous year, probably due to the intensification of the waves of the pandemic (Figure 7). It was observed that in Bulgaria and Romania, the tide of COVID-19 deaths during the autumn of 2021 was stronger than before, even though the number of fully vaccinated people was also increasing.

**Figure 8:**
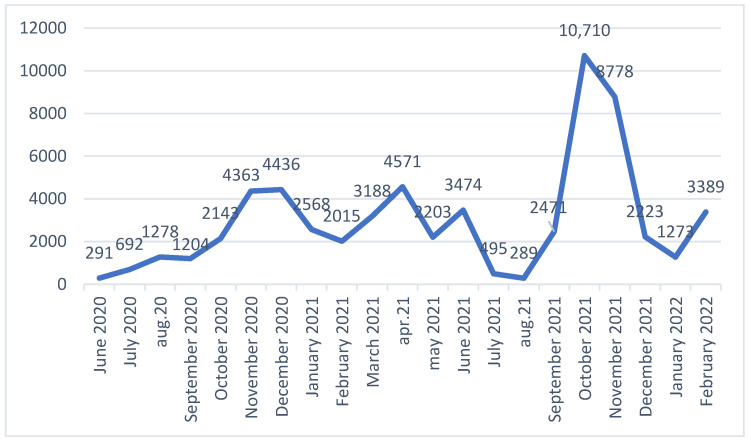
Number of deaths due to COVID-19 from June 2020 to March 2022, by month.

**Figure 9:**
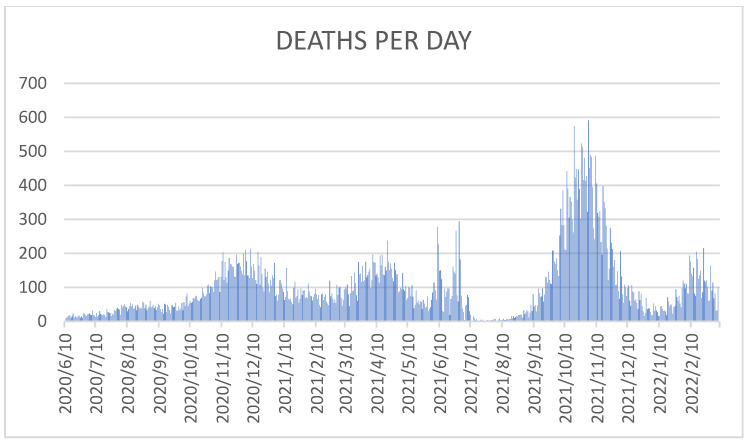
Number of deaths due to COVID-19 in Romania, by day (from June 2020 to December 2021).

Figure 8 and Figure 9 are graphical representations of the number of deaths due to COVID-19 during the state of emergency in Romania (by day and month, from June 2020 to March 2022). The highest number of deaths in Romania was registered on 2 November 2021, when 591 people died due to SARS-CoV-2 infection. Analyzing the images above, it appears that the waves of the pandemic had an increasing influence on the number of deaths at the national level, respectively. The number of deaths due to COVID-19 strongly contributed to the considerable increase in the total number of deaths nationwide.

After the peak in 2021, the data show a significant decrease in the number of deaths in 2022 and 2023, especially in the middle and elderly age groups (Table 1).

This decline can be attributed to efforts to mitigate the pandemic and the implementation of prevention and vaccination measures that have greatly reduced the incidence and mortality associated with COVID-19. The topic of vaccination was another interesting subject, because of the numerous studies showing that vaccination rate vastly affects mortality. In a study conducted by Sobczak [6], it was shown that mortality decreased in the countries with a higher number of distributed vaccine doses, booster vaccine doses and fully vaccinated people.

However, this was not a common trend. In some countries, such as Bulgaria, Latvia, Lithuania and Romania, the number of fully vaccinated people positively correlated with the mortality rate; whereas, in Hungary, Romania and Slovakia, the numbers of vaccine doses and boosters increased the mortality rate of COVID-19 [5].

Another interesting trend observed is that the daily number of vaccinations per million coincided with the number of daily deaths per million in Romania, Hungary and Slovakia [6]. This can be related to a common awareness of the dangers of COVID-19 fatality, which led people to protect themselves through vaccination.

### 3.5. Romania and the European Countries During the COVID-19 Pandemic

**Figure 10:**
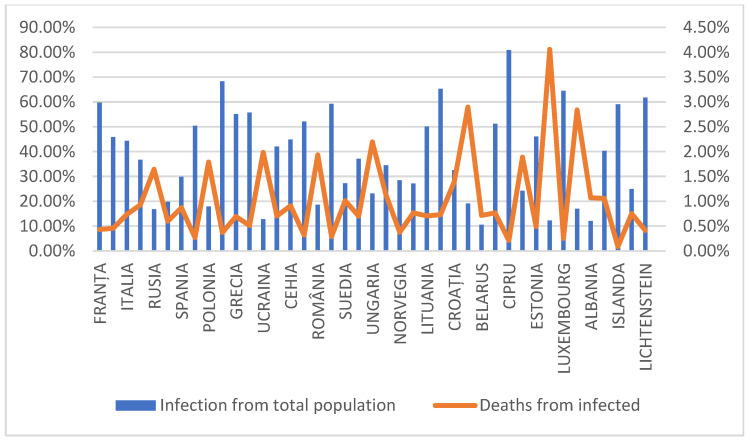
Share of infection cases and deaths from total infected population (countries in the European area).

Referring to the share of infections from the total population in the European area, the countries that recorded the highest percentages are Cyprus, Austria, Slovenia, Luxembourg and Liechtenstein. Despite having recorded an increased percentage of infections, the countries are at the bottom of the ranking regarding the percentage of deaths due to COVID-19 in the total population. Regarding the percentage of deaths in the total infected population, Bosnia and Herzegovina (4.06%), Bulgaria (2.9%), Macedonia (2.84%), Hungary (2.20%) and Ukraine (1.98%) are the five countries with the highest rates. Romania ranks sixth highest on this metric, with 1.93% deaths in the total infected population (and Figure 10). The increased mortality of the infected population can be related to the inefficient response of the public health system and government to the COVID-19 crisis.

In order to establish the relation between the share of infections and the share of deaths in the total infected population, we analyzed the data using a Pearson correlation (Table 4).

The results of the analysis showed that there is a strong negative correlation between the two variables, meaning that the share of deaths is higher among the countries with a low infection share (Table 5). For example, as shown in Figure 8 and Table 6, we concluded that the countries with a share of infection under 20% and a higher number of deaths are Russia, Poland, Ukraine, Romania, Belarus, Bosnia and Herzegovina and Albania. This is an interesting observation, and unfortunately it reflects complex realities related to healthcare systems, access to adequate treatments and government responses to the COVID-19 pandemic. In many of these countries (notably Ukraine, Russia, Belarus and Bosnia), healthcare systems were already under strain before the pandemic. In these countries, access to quality healthcare and the resources needed to deal with a public health crisis like COVID-19 were limited.

Otherwise, an unhealthy diet and the lack of an active lifestyle are common problems in many of these countries, which can lead to a low body resistance to infectious diseases, including COVID-19. These factors can contribute to a higher prevalence of comorbidities such as cardiovascular disease, diabetes or chronic respiratory diseases, meaning a higher risk of severe forms of infection and death. Smoking and alcohol abuse are also much more prevalent in some of these countries, and these behaviors are important risk factors for the severe course of COVID-19 [6].

Another cause of the lower number of infection declared in these countries can be the testing rate, late diagnosis and treatment. In many cases, COVID-19 infections were not diagnosed in time or did not receive appropriate treatment, especially for those in the vulnerable categories. Also, some of these countries have struggled to implement effective testing strategies.

The vaccination process has been slower in some regions, and vaccine hesitancy has also been a factor contributing to the higher death rate. For example, in Russia and Ukraine, there have been controversies surrounding vaccines, and in Belarus and Bosnia, there has been a negative perception of government interventions, which has led to lower vaccination coverage. Besides the vaccination policy, proper health-related education seems to be relevant in terms of the vaccination ratio, as people not educated in medical sciences tended to be less likely to be vaccinated than people with a medical background. According to the study by Walkowiak et al. [7], in most EU countries, the vaccination rate reaches 75%, except Eastern Bloc countries. In the case of the Eastern Bloc, the value is much lower, whereas the lowest rate can be observed in Bulgaria and Romania. By a comparative analysis of vaccination policy in Poland and Lithuania, the authors assumed that the introduction of vaccine certificates and extensive restrictions for unvaccinated citizens successfully increased the vaccination rate in Lithuania.

In some of these countries, the underreporting of COVID-19 cases, or even deaths, may be a problem. In particular, in Russia and Ukraine, there are suspicions that the actual number of cases and deaths was higher than officially reported, due to a flawed reporting system or political motivation to minimize the impact of the pandemic. In addition, in Bosnia and Albania, due to a more fragile healthcare system and the difficulty of collecting accurate data, the actual number of deaths is likely to be higher than that reflected in official statistics.

Public awareness of measures to prevent the spread of the virus (such as wearing masks, social distancing, frequent hand hygiene, etc.) was often insufficient. In some of these countries, information campaigns were less effective and resistance to lockdown measures was higher.

Thus, although the percentage of infections may be relatively low compared to the total population, the higher mortality rate may be due to a combination of factors, such as an inefficient health system, a high prevalence of comorbidities and, potentially, a lack of adequate medical infrastructures and education for health, insufficient vaccination and underreporting of cases. There may also be a significant delay in diagnosis and treatment, which affects the chances of recovery for patients and increases the risk of death.

According to Table 5 and Appendix A, another aspect that can be observed is that the European countries with the lowest life expectancy at birth (Estonia, Lithuania, Latvia, Poland, Slovakia, Hungary, Romania and Bulgaria) although are not in the top of the countries with the highest percentage of infections from the total population, five out of eight are in the top 15 countries with the highest percentage of deaths from the total infected population (Table 5). The countries with the highest life expectancy (Spain, Switzerland, France, Italy, Belgium, Norway, Sweden, Liechtenstein, Malta) are in the second half of the ranking, with a percentage of deaths in the infected population below 1.01% (Appendix A). This phenomenon reflects the vulnerabilities related to the pandemic, and the general health status of the population, economic conditions, access to healthcare and the efficiency of health systems. Thus, by comparing these indicators, we can observe how factors such as insufficient health infrastructure, widespread comorbidities and implemented preventive measures influence not only the impact of the COVID-19 crisis, but also the long-term health prospects of the population.

Therefore, in analyzing the impact of the COVID-19 pandemic on older age groups, it is important to consider the correlation between the share of deaths caused by the virus and life expectancy at birth. Life expectancy at birth represents the average number of years a person born in a given year in a specific country can expect to live, assuming that the age-specific mortality rates remain constant throughout their lifetime. Higher life expectancy reflects progress in healthcare, but is also a sign of an aging population, which can put pressure on healthcare systems and social services. Life expectancy at birth is a key public health indicator that reflects the overall health conditions of a population, including access to healthcare, nutrition and living conditions. For the data we have, we analyzed the correlation between life expectancy at birth (Appendix A) and the share of deaths from the total population among 27 EU states. The results of the Pearson correlation shows that there is a good negative correlation between variables (*p* = 0.000) with r = −0.649, meaning that the share of deaths is lower among the countries with high life expectancy (Table 6). This means that a well-established health system and a good education system can have a huge impact on the healthy aging of a population, and can counteract the devastating effects that a pandemic can have on an aging population.

### 3.6. Worldwide Pandemic Situation

**Figure 11:**
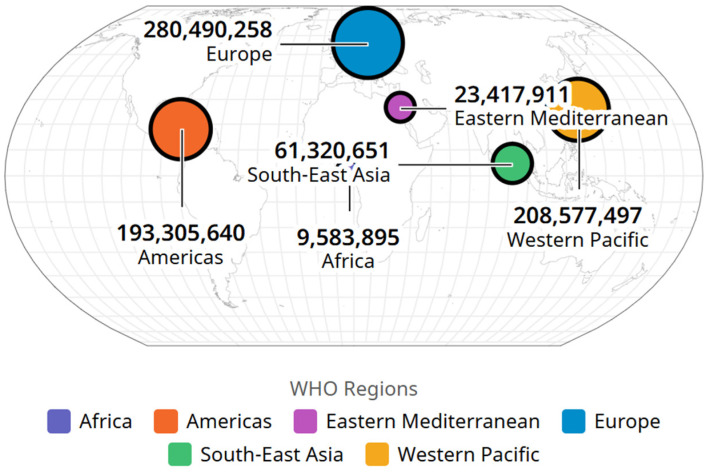
SARS-CoV-2 infections by WHO regions, image from WHO site, https://data.who.int/dashboards/covid19/deaths?m49=958&n=o (accessed on 8 November 2024).

**Figure 12:**
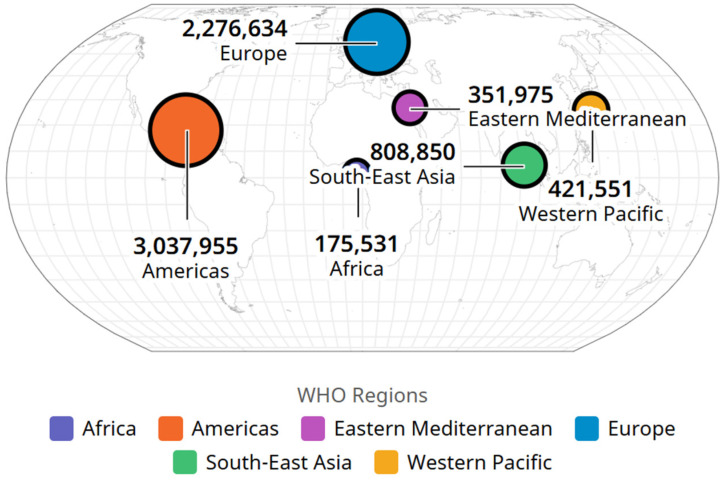
Deaths due to SARS-CoV-2 by WHO regions, image from WHO site, accessed on https://data.who.int/dashboards/covid19/deaths?m49=958&n=o (accessed on 8 November 2024).

The WHO reported that until November 2024, there were 776.696.616 cases of COVID-19 (Figure 11) and 7,072,509 deaths registered worldwide (Figure 12).

According to Figure 9 and Figure 10, using data from the World Health Organization website, the largest reservoir of COVID-19 cases was represented by the European region (280.490.2580 infections) followed by the Western Pacific region (208.577.497 infections) and the Americas region (193.305.640 infections) (Table 7).

The number of deaths caused by COVID-19, however, follows a different distribution, with America ranking first (3,037,955 deaths), followed by the European region (2,276,634 deaths) and the southeast Asian region (808,850), according to Table 8.

In a public document, the World Health Organization has classified a series of pathologies that, according to the studies, predispose the patient both to the risk of infection with the SARS-CoV-2 virus, and to a higher risk of death from the COVID-19 disease [8]. A lot of studies have correlated older age and multiple comorbidities with increased hospital length of stay, intensive care unit admission and increased mortality among the infected population [9]. In 2020, Roncon et al. concluded that people with diabetes were three times more likely to experience severe disease symptoms or death from COVID-19 [10]. Also, hypertension, cardiovascular and cerebrovascular diseases increase the chances of developing a severe form of COVID-19 disease. A meta-analysis which included studies with 29.909 infected patients and 1.445 deaths concluded that advanced age (over 65 years), male gender, hypertension, cardiovascular disease, diabetes, chronic obstructive disease and malignant tumors were associated with a higher risk of death due to SARS-CoV-2 infection [11]. Data from around the world suggest that age itself is the most significant risk factor for severe COVID-19 and adverse health effects.

Overweight and obesity are chronic metabolic conditions that increase the risk of high blood pressure, type 2 diabetes, coronary heart disease, stroke and certain types of cancer. These pathologies are on the list of comorbidities that can modify the host’s response to the disease, predisposing the patient to an increased risk of death as a result of an infection with SARS-CoV-2 and other conditions. Thus, a pre-emergent condition for the appearance of comorbidities is obesity. America and Europe are the top two places for average body mass index (reported by WHO in 2017; Table 9).

This may be related to the increased number of deaths recorded, given that the average values of BMI in Europe and America register values specific to being overweight.

In percentage terms, the highest mortality rates from the total number of infected people was recorded in Africa (Table 10). COVID-19 deaths were apparently under-counted early in the pandemic, and continue to be under-counted in several countries, especially in Africa, while over-counting probably currently occurs in several other countries, especially those with intensive testing [12]. However, compared to the total population in the territory, the number of reported infections was small, and sub-Saharan Africa reported relatively few COVID-19 deaths [13]. In many African countries, the capacity to test and report cases has been limited compared to more developed regions. This makes the official number of reported cases lower. The increased mortality can also be attributed to the lack of access to medical services and inadequate hygiene conditions. Many African countries face serious gaps in health infrastructure, with understaffed hospitals, insufficient medical staff and limited resources. These deficiencies make treatments for serious illnesses, including COVID-19, less effective and survival less likely.

## 4. Discussion

The year 2021 was a turning point in terms of mortality in Romania, with the highest number of deaths recorded since 1938. This increase can be largely attributed to the effects of the COVID-19 pandemic, meaning that life during the pandemic was not the same as before.

A study by Sharma et al. shows that the risk of death from COVID-19 is about 20% higher among men, which is in line with the trends also observed in Romania [1]. This may suggest an increased vulnerability of men to the disease, and also a more frequent tendency to develop serious complications following infection with COVID-19, requiring admission to intensive care units and the use of mechanical ventilation. This emphasizes the importance of faster and more effective interventions for this population group, as well as the need for personalized prevention and treatment strategies. Differences in mortality between genders, both before and during the pandemic, point to the need to direct medical resources to more vulnerable groups, such as men, who appear to be at higher risk from infectious diseases such as COVID-19.

In Romania, the highest mortality rate was recorded in the 70–79 age group, followed by the over 80 age group and the 60–69 age group. A meta-analysis of 14 relevant publications, which included 29,909 infected patients and 1445 deaths, summarized the conclusions regarding the association between age, gender, comorbidities and the risk of death due to SARS-CoV-2 infection. It was concluded that advanced age (over 65 years), male gender, hypertension, cardiovascular disease, diabetes, chronic obstructive disease and malignant tumors were associated with a higher risk of death due to SARS-CoV-2 infection [6]. After our analysis, we also concluded that older age groups had a significantly higher number of deaths compared to younger age groups, confirming that older age is an important risk factor of death due to COVID-19.

The higher incidence among older age groups can be attributed to the aging process itself, because all physiological functions slow down and the body and organs change. Blood vessels become more rigid, the will no longer pumps blood as efficiently and hypertension or various other cardiovascular diseases may occur. Cognitive function no longer fulfill its role as well as at younger ages, cognitive processes may slow down and problems with memory and concentration can appear. The immune system becomes weaker, making it easier for infections and diseases to develop. The musculoskeletal system is also affected by the aging process, which increases susceptibility to injury. It is also well-known that with age comes a natural, physiological decline in respiratory function. Decreased muscle strength and decreased lung elasticity are just two of the factors that will lead to a decreased breathing capacity. All these changes suggest why an elderly person is more susceptible to both SARS-CoV-2 infection and a high risk of death.

Although exercise can not directly prevent SARS-CoV-2 infection, it is well-known that it is an effective prevention method by strengthening immunity, reducing inflammation and improving general health, which can have a significant positive impact on post-COVID-19 recovery and reducing the risks of severe complications. Physical exercise programs adapted to the needs of the elderly could play a crucial role in the effective management of pandemics and in protecting the vulnerable groups.

In Romania and in countries facing similar situations, campaigns to promote, for example, frequently medical checks of men should be implemented in order to detect underlying health conditions that exacerbate SARS-CoV-2 infection response, such as cardiovascular disease, respiratory or metabolic diseases.

In countries with a growing elderly population, it is important to improve access to health services and vaccination, and to enhance protective care measures in elderly homes.

For future potential health crises, it is important to prioritize vulnerable groups, such as males and those in higher age categories, with regard to ICU beds, ventilators and other critical care resources. Public health policies should not only focus on disease itself, but also on the health inequalities that contribute to the higher mortality rates in this specific population, such as improving cardiovascular health (physical exercise programs prevention), smoking cessation programs and increasing the percentage of budgets on healthcare access, especially in rural and underserved areas.

Prior to the pandemic, circulatory diseases were the leading cause of death in Romania, but during the pandemic, respiratory diseases emerged as the dominant factor, reflecting the direct and significant impact of COVID-19. The COVID-19 pandemic led to a significant rise in the number of deaths from respiratory diseases. In 2020, deaths from respiratory conditions more than doubled, largely due to the high incidence of COVID-19 infections, accounting 13.0% from total deaths. This trend peaked in 2021 at 19.5%, underlining the severe public health crisis in progress. However, in 2022, as COVID-19 infections and other major respiratory diseases declined, the proportion of deaths from respiratory diseases decreased to 10.9%.

**Figure 13:**
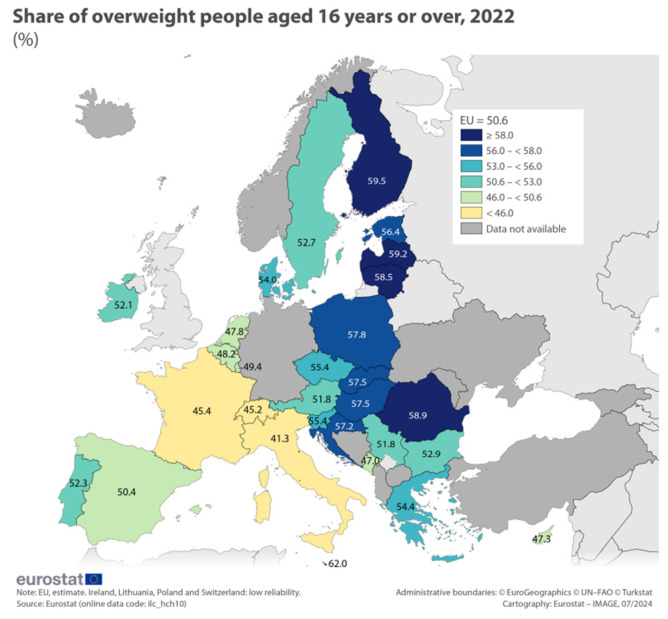
Share of overweight people aged 16 years or over, 2022.

Cardiovascular diseases, mentioned above as being the leading cause of death in Romania before the pandemic, are a main consequence of overweight and obesity. Analyzing the BMI trends in the European countries, it seems that Romania has a higher share of overweight people aged 16 years and over (Figure 13).

Regarding the causality and seasonality of deaths, the analyzed periods reflect a complex interaction between demographic factors, seasonality and the effects of the pandemic on population health. These facts emphasize the importance of the continuous monitoring of mortality and causes of death for a thorough understanding of public health, with a view to adopting effective disease prevention and management policies.

During the pandemic, a lot of countries implemented measures in order to prevent the spread of the virus, such as wearing masks, travel restrictions, working from home and proper hygiene [14]. Governments introduced lockdowns and encouraged public areas sanitizing to reduce the spread of SARS-CoV-2 [15]. The analysis of the above countries’ governmental responses showed that the stringency of the measuresdecreased the mortality rate just in Romania, but increased mortality in countries such as the Czech Republic, Hungary, Lithuania, Poland and Slovakia. This positive correlation was explained by the fact that restrictions were introduced when higher numbers of infection occurred, a period which also reflected a higher number of deaths [6]. Megarbane et al. proposed that lockdowns are the most important measure, and should be considered as a standard countermeasure in future epidemics [16]. Nevertheless, the main measure that changed the course of the COVID-19 pandemic was vaccination, which prevented the deaths of millions of people. Based on the reported COVID-19 deaths, global analysis estimated that the first year of COVID-19 vaccination prevented about 14.4 million COVID-19 deaths, whereas according to excess mortality data, vaccination prevented 19.8 million deaths [17].

A factor related to lower vaccination rates in at least some Eastern European countries might be related to a relatively high percentage of citizens living in secluded communities, such as the Roma community. It has been reported that even before the pandemic, members of the Roma community strayed from vaccine administration as they had impaired access to the healthcare [18].

Besides the vaccination policy, proper health-related education seem to be relevant in terms of vaccination uptake levels, as people not educated in medical sciences tended to be less likely to be vaccinated than people with a medical background.

## 5. Conclusions

A significant higher number of deaths were registered for males compared to women in Romania, a trend that aligns with global tendencies.

The risk of death from COVID-19 increases significantly with age, meaning that it is important to prioritize vulnerable groups and to adapt public health policies in order to not only focus on curing diseases, but also on prevention and primary prophylaxis of the potential underlying health conditions that can lead to an exacerbated response after a COVID-19 infection. The health status of the population, which is also a factor contributing to an increased mortality risk related to an infectious disease should also be addressed. This can be possible by improving access to physical activities, by creating specially designed places for leisure activities, implementing nutritional education, implementing smoking and alcohol cessation campaigns and increasing the percentage of country budgets on healthcare access, especially in rural and underserved areas.

In the pandemic years, deaths from respiratory diseases increased considerably, from 6.8% in 2019 to 19.5% in 2021, which was directly related to the increased incidence of COVID-19.

We concluded that the countries with a share of infection under 20% from the total population have a higher number of deaths from the number of the total infected. The higher COVID-19 mortality in Central and Eastern European countries might be caused by the overall healthcare quality. According to the data published by Eurostat, central/eastern European countries tend to spend a lower percentage of their budgets on healthcare compared to Western countries [19]. Moreover, many of those countries have had to deal with multiple reforms of the healthcare system after the fall of the USSR (Union of Soviet Socialist Republics), which for economically weaker countries, might remain a problem [20].

Factors such as cardiovascular disease, diabetes or chronic respiratory diseases can contribute to a higher prevalence of comorbidities and an automatically higher risk of death. Smoking and alcohol abuse are also behaviors that increase the risk for a severe course of COVID-19. This emphasizes the importance of nutritional and health education among the population.

The higher mortality recorded in countries with low infection shares may be due to a combination of factors, such as an inefficient health system, the prevalence of comorbidities, a lack of adequate medical resources and education for health, insufficient vaccination and the underreporting of cases. There may also be a significant delay in diagnosis and treatment, which affects the chances of recovery for patients and increases the risk of death.

Higher life expectancies reflects progress in healthcare, but are also a sign of aging populations, which can put pressure on healthcare systems and social services. Related to the impact of older age on the course of COVID-19, it is important to underline that a well-established health system and a good education can have a huge impact on healthy aging and can counteract the devastating effects that a pandemic can have on an aging population.

The largest reservoir of COVID-19 cases was represented by the European region (280,490,2580 infections). Numerically, however, most deaths occurred in America (3,037,955 deaths), followed by the European region (2,276,634 deaths). America and Europe are the top two places ranked by average body mass index (reported by WHO). This may be related to the increased number of deaths recorded, given that the average values of BMI in Europe and America register values specific to being overweight.

## Figures and Tables

**Table 4:** Pearson correlation between share of infection and share of deaths in 27 EU countries.

		Deaths in Total Infected Population
Infection in total population	Pearson correlation	−0.720
	sig. (two-tailed)	0.000
	N	27

**Table 5:** Share of infection cases, deaths from total infected population and deaths from total population (countries in the European region).

	Country	Total Population	Cases	Deaths	Share of Infection from Total Population	Share of Deaths from Infected Population	Share of Deaths from Total Population
1	Russia	145,940,812	24,547,989	403,508	16.82%	1.64%	0.28%
2	Turkey	85,751,852	17,004,728	101,419	19.83%	0.60%	0.12%
3	Germany	83,783,942	38,437,756	174,979	45.88%	0.46%	0.21%
4	United Kingdom	68,102,788	25,006,869	232,112	36.72%	0.93%	0.34%
5	France	65,360,889	39,023,328	168,091	59.70%	0.43%	0.26%
6	Italy	60,408,345	26,826,486	197,542	44.41%	0.74%	0.33%
7	Spain	46,765,702	13,980,340	121,852	29.89%	0.87%	0.26%
8	Ukraine	43,576,860	5,541,734	109,923	12.72%	1.98%	0.25%
9	Poland	37,821,665	6,755,185	120,875	17.86%	1.79%	0.32%
10	Romania	19,161,134	3,566,466	68,899	18.61%	1.93%	0.36%
11	Netherlands	17,157,809	8,644,223	22,986	50.38%	0.27%	0.13%
12	Belgium	11,620,241	4,888,331	34,339	42.07%	0.70%	0.30%
13	Greece	10,392,597	5,724,778	39,606	55.09%	0.69%	0.38%
14	Czech Republic	10,720,994	4,807,669	43,660	44.84%	0.91%	0.41%
15	Portugal	10,178,868	5,669,374	29,018	55.70%	0.51%	0.29%
16	Sweden	10,137,607	2,764,353	27,982	27.27%	1.01%	0.28%
17	Hungary	9,645,622	2,235,887	49,084	23.18%	2.20%	0.51%
18	Belarus	9,447,450	994,038	7118	10.52%	0.72%	0.08%
19	Austria	8,902,600	6,082,821	22,534	68.33%	0.37%	0.25%
20	Switzerland	8,586,550	4,466,918	14,170	52.02%	0.32%	0.17%
21	Bulgaria	7,000,039	1,337,252	38,743	19.10%	2.90%	0.55%
22	Serbia	6,963,764	2,583,470	18,057	37.10%	0.70%	0.26%
23	Denmark	5,814,461	3,442,484	9919	59.21%	0.29%	0.17%
24	Finland	5,522,848	1,499,712	11,466	27.15%	0.76%	0.21%
25	Slovakia	5,450,421	1,883,245	21,247	34.55%	1.13%	0.39%
26	Norway	5,345,599	1,523,402	5732	28.50%	0.38%	0.11%
27	Croatia	4,130,304	1,347,441	18,774	32.62%	1.39%	0.45%
28	Bosnia and Herzegovina	3,301,000	403,890	16,400	12.24%	4.06%	0.50%
29	Albania	2,802,471	337,192	3608	12.03%	1.07%	0.13%
30	Lithuania	2,793,471	1,398,560	9847	50.07%	0.70%	0.35%
31	Republic of Moldova	2,681,735	650,542	12,280	24.26%	1.89%	0.46%
32	Slovenia	2,084,301	1,359,672	9914	65.23%	0.73%	0.48%
33	Macedonia	2,077,132	352,032	9990	16.95%	2.84%	0.48%
34	Latvia	1,912,000	977,765	7475	51.14%	0.76%	0.39%
35	Estonia	1,324,820	610,471	2998	46.08%	0.49%	0.23%
36	Cyprus	875,900	708,559	1492	80.89%	0.21%	0.17%
37	Montenegro	622,359	251,280	2654	40.38%	1.06%	0.43%
38	Malta	493,559	123,114	925	24.94%	0.75%	0.19%
39	Iceland	357,000	210,656	186	59.01%	0.09%	0.05%
40	Liechtenstein	35,000	21,603	89	61.72%	0.41%	0.25%
41	Luxembourg	613,894	395,802	1000	64.47%	0.25%	0.16%

**Table 6:** Pearson correlation between life expectancy at birth and share of deaths in 27 EU countries.

		Share of Deaths from Total Population
Life expectancy	Pearson correlation	−0.649
	sig. (two-tailed)	0.000
	N	27

**Table 7:** Number of SARS-CoV-2 cases, by WHO areas.

	Area	SARS-CoV-2 Infections
1	Europe	280,490,258
2	Western Pacific	208,577,497
3	America	193,305,640
4	Southeast Asia	61,320,651
5	Eastern Mediterranean	23,417,911
6	Africa	9,583,895

**Table 8:** Number of SARS-CoV-2 deaths, by WHO regions.

	Area	SARS-CoV-2 Deaths
3	America	3,037,955
1	Europe	2,276,634
4	Southeast Asia	808,850
2	Western Pacific	421,551
5	Eastern Mediterranean	351,975
6	Africa	175,531

**Table 9:** Average body mass index, by WHO regions.

Mean BMI 18 Years +			
WHO Areas	Both Sexes	Male	Female
America	27.6	27.4	27.8
Europe	26.4	26.7	26.1
Eastern Mediterranean	26.1	25.3	27
Western Pacific	23.7	24	23.4
Africa	23.2	22.4	24.1
Southeast Asia	22.2	22	22.4

**Table 10:** Death rate from total infected by WHO regions.

	WHO Areas	Infection Number	Deaths Number	Share of Deaths from Infections
6	Africa	9,583,895	175,531	1.83%
3	America	193,305,640	3,037,955	1.57%
5	Estern Mediterranean	23,417,911	351,975	1.50%
4	Southeast Asia	61,320,651	808,850	1.32%
1	Europe	280,490,258	2,276,634	0.81%
2	Western Pacific	208,577,497	421,551	0.20%

**Figure A1:**
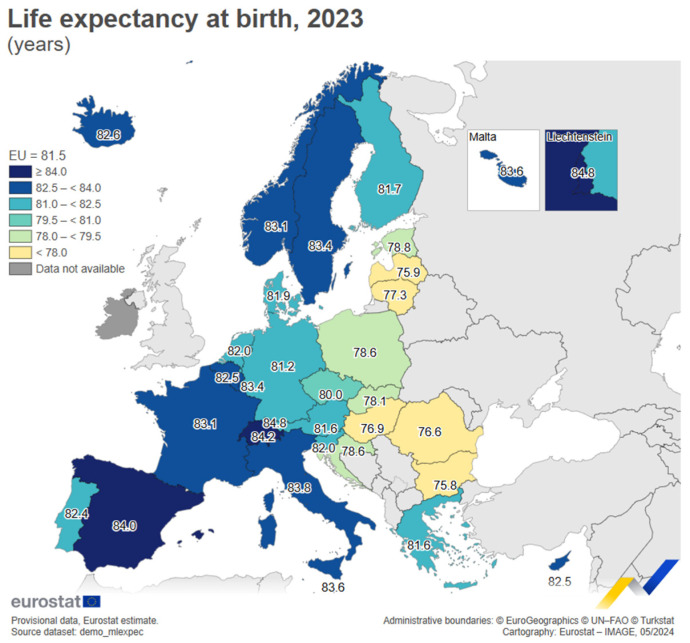
Image from EUROSTAT site: https://ec.europa.eu/eurostat/web/products-eurostat-news/w/ddn-20240503-2#:~:text=In%2015%20countries%2C%20life%20expectancy,)%20and%20Romania%20(76.6, accessed on 8 November 2024, 08:15.

## Data Availability

This report is using public data that does not contain personally identifiable information. Ethical approval is not applicable. The data are already anonymized or aggregated to protect privacy.

## Abbreviations

The following abbreviations are used in this manuscript:

EU	European Union
BMI	Body mass index
WHO	World Health Organization
NIS	National Institute of Statistics
MIA	Ministry of Internal Affairs

## References

[1] Sharma G., Volgman A.S., Michos E.D. (2020). Sex Differences in Mortality From COVID-19 Pandemic: Are Men Vulnerable and Women Protected?. JACC Case Rep..

[2] Williamson E.J., Walker A.J., Bhaskaran K., Bacon S., Bates C., Morton C.E., Curtis H.J., Mehrkar A., Evans D., Inglesby P. (2020). Factors associated with COVID-19-related death using OpenSAFELY. Nature.

[3] World Population Prospects—Population Division—United Nations [Internet]. https://population.un.org/wpp/.

[4] Chen N., Zhou M., Dong X., Qu J., Gong F., Han Y., Qiu Y., Wang J., Liu Y., Wei Y. (2020). Epidemiological and clinical characteristics of 99 cases of 2019 novel coronavirus pneumonia in Wuhan, China: A descriptive study. Lancet.

[5] The Lancet (2020). The gendered dimensions of COVID-19. Lancet.

[6] Sobczak M., Pawliczak R. (2022). COVID-19 mortality rate determinants in selected Eastern European countries. BMC Public Health.

[7] Walkowiak M.P., Walkowiak J.B., Walkowiak D. (2021). COVID-19 Passport as a Factor Determining the Success of National Vaccination Campaigns: Does It Work? The Case of Lithuania vs. Poland. Vaccines.

[8] WHO Site. https://www.who.int/docs/default-source/ncds/un-interagency-task-force-on-ncds/uniatf-policy-brief-ncds-and-covid-030920-poster.pdf?ua=1.

[9] Richardson S., Hirsch J.S., Narasimhan M., Crawford J.M., McGinn T., Davidson K.W., Barnaby D.P., Becker L.B., Chelico J.D., the Northwell COVID-19 Research Consortium (2020). Presenting Characteristics, Comorbidities, and Outcomes Among 5700 Patients Hospitalized with COVID-19 in the New York City Area. JAMA.

[10] Roncon L., Zuin M., Rigatelli G., Zuliani G. (2020). Diabetic patients with COVID-19 infection are at higher risk of ICU admission and poor short-term outcome. J. Clin. Virol. Off. Publ. Pan Am. Soc. Clin. Virol..

[11] Wang B., Li R., Lu Z., Huang Y. (2020). Does comorbidity increase the risk of patients with COVID-19: Evidence from meta-analysis. Aging.

[12] Parohan M., Yaghoubi S., Seraji A., Javanbakht M.H., Sarraf P., Djalali M. (2020). Risk factors for mortality in patients with Coronavirus disease 2019 (COVID-19) infection: A systematic review and meta-analysis of observational studies. Aging Male Off. J. Int. Soc. Study Aging Male.

[13] Ioannidis J.P.A. (2021). Over- and under-estimation of COVID-19 deaths. Eur. J. Epidemiol..

[14] Girum T., Lentiro K., Geremew M., Migora B., Shewamare S., Shimbre M.S. (2021). Optimal strategies for COVID-19 prevention from global evidence achieved through social distancing, stay at home, travel restriction and lockdown: A systematic review. Arch. Public Health = Arch. Belg. Sante Publique.

[15] Onyeaka H., Anumudu C.K., Al-Sharify Z.T., Egele-Godswill E., Mbaegbu P. (2021). COVID-19 pandemic: A review of the global lockdown and its far-reaching effects. Sci. Prog..

[16] Mégarbane B., Bourasset F., Scherrmann J.M. (2021). Is Lockdown Effective in Limiting SARS-CoV-2 Epidemic Progression?—A Cross-Country Comparative Evaluation Using Epidemiokinetic Tools. J. Gen. Intern. Med..

[17] Watson O.J., Barnsley G., Toor J., Hogan A.B., Winskill P., Ghani A.C. (2022). Global impact of the first year of COVID-19 vaccination: A mathematical modelling study. Lancet Infect. Dis..

[18] Sándor J., Vincze F., Shrikant M.L., Kőrösi L., Ulicska L., Kósa K., Ádány R. (2022). COVID-19 vaccination coverage in deprived populations living in segregated colonies: A nationwide cross-sectional study in Hungary. PLoS ONE.

[19] Healthcare Expenditure Statistics [Internet]. https://ec.europa.eu/eurostat/statistics-explained/index.php?title=Healthcare_expenditure_statistics.

[20] Romaniuk P., Szromek A.R. (2016). The evolution of the health system outcomes in Central and Eastern Europe and their association with social, economic and political factors: An analysis of 25 years of transition. BMC Health Serv. Res..

